# Waterborne Polyurethanes as a New and Promising Class of Kinetic Inhibitors for Methane Hydrate Formation

**DOI:** 10.1038/s41598-019-46274-w

**Published:** 2019-07-05

**Authors:** Abdolreza Farhadian, Arman Kudbanov, Mikhail A. Varfolomeev, Didier Dalmazzone

**Affiliations:** 1 Department of Physical Chemistry, Kazan Federal University, Kremlevskaya str. 18, 420008 Kazan, Russian Federation; 2 UCP, ENSTA ParisTech, Université Paris-Saclay, 828 Boulevard des Maréchaux, 91762 Palaiseau, Cedex France

**Keywords:** Thermodynamics, Polymer synthesis, Natural gas

## Abstract

A facile, new and promising technique based on waterborne polymers for designing and synthesizing kinetic hydrate inhibitors (KHIs) has been proposed to prevent methane hydrate formation. This topic is challenging subject in flow assurance problems in gas and oilfields. Proposed technique helps to get KHIs with required number and distance of hydrophilic and hydrophobic groups in molecule and good solubility in water. The performance of these new KHIs was investigated by high pressure micro-differential scanning calorimeter (HP-μDSC) and high-pressure autoclave cell. The results demonstrated the high performance of these inhibitors in delay the induction time (10–20 times) and reduce the hydrate growth rate (3 times). Also they did not increase hydrate dissociation temperature in comparison with pure water and show thermodynamic inhibition as well. Inhibition effect of synthesized polymers is improved with the increase of concentration significantly. Since this is the first report of the use of waterborne polymers as kinetic hydrate inhibitor, we expect that KHIs based on waterborne-based polymers can be a prospective option for preventing methane hydrate formation.

## Introduction

**Figure 1:**
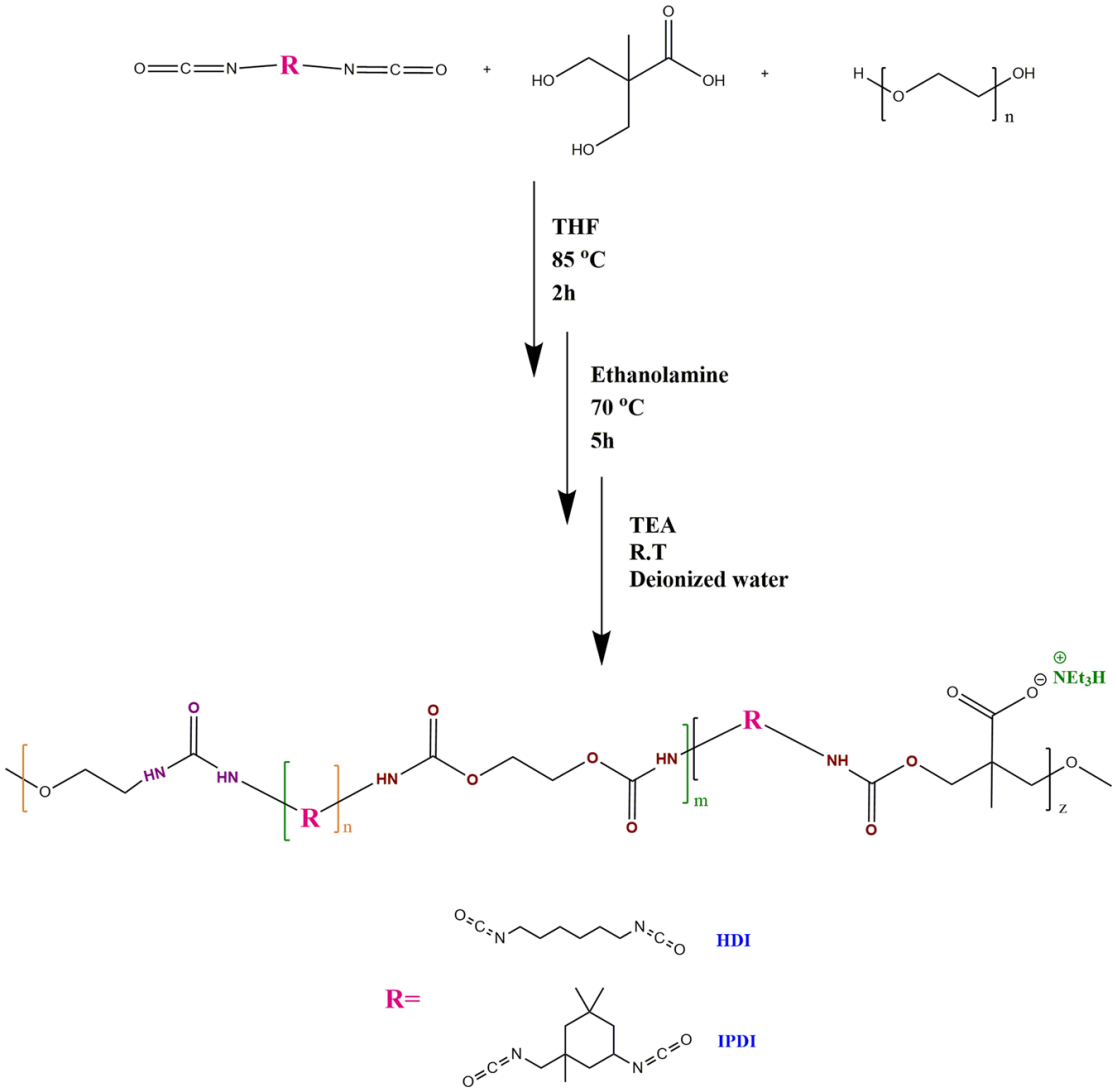
Synthesis of waterborne poly urea/urethanes (WPUUs).

Undoubtedly, one of the most important challenges in the design/synthesis and performance evaluation of gas hydrate inhibitors is their solubility in water, particularly since it had been demonstrated that hydrates cause blockages of hydrocarbon pipelines (1933–1944)^1^. High pressure, low temperature, water and guest molecules like light hydrocarbons, such as methane, ethane and propane provide conditions for the formation of non-stoichiometric crystalline solid compounds, called gas hydrates (or/also clathrates). Gas hydrates are attractive objects for researchers, not only because of their negative aspects in oil and gas production. For instance, these compounds will be beneficial when they are used as energy carriers in the future. Moreover, they could be used in desalination, refrigeration, gas storage and gas separation^2–10^. In general, these applications take advantage of the hydrates formation and decomposition cycles. Thus, some researchers have focused on the synthesis and design of promoters for creating mild conditions of crystallization or increasing the kinetics of hydrate formation, in order to reduce process expenses^11,12^. On the other hand, the formation of gas hydrates can give rise to several problems, like flow assurance issues and plugging in hydrocarbon pipelines and their processing equipment. Using chemical reagents is one of the best ways to effectively deal with this problem. The most widely used among them are thermodynamic hydrate inhibitors (THIs) such as methanol, ethylene glycol and etc. These reagents decrease the temperature of gas hydrate formation. However, THIs are only efficient at high concentrations. This leads to the high cost of their use and causes environmental risks. The main alternative of THIs are low-dosage hydrate inhibitors (LDHIs)^13^. LDHIs can be divided into two major categories: kinetic hydrate inhibitors (KHIs) and anti-agglomerants (AAs). They have received increasing attention in both academic and industrial fields because of their better economic and environmental performances. KHIs present water-soluble substances (usually polymers) that help to delay effectively the hydrate formation in time to ensure transport of the fluid without blocking of pipelines^14^. Many categories of chemicals, such as various types of poly-*N*-vinyllactam, hyperbranched poly(esteramide)s, poly-ester pyroglutamates, maleic copolymers, alkylamide derivatives, acrylamide, amine oxide derivatives polyaspartamides, modified starch, starch derivatives, polymeric hydrogels and amino acids were investigated as KHIs^13–16^. AAs are mainly surfactants and convert hydrates to non-sticky and transferable slurry. In the literature, there is evidence that AAs have better performance than KHIs in some conditions such as higher subcooling or longer transportation distances^17,18^. In previous reports^19–22^, it has been proved that any KHI with a hydrophilic tail and/or hydrophobic section interacts with water molecules. As a result, the hydrogen bonds network in aqueous solution is disturbed and the hydrate formation mechanism is slowed down. Moreover, if the KHI has functional groups such as aromatic or cyclic structures and alkyl chains in its hydrophobic part, it can interact with water molecules more than other KHIs without any functional groups. Because of a stronger steric hindrance effect, the inhibition effect is then increased, provided that the water solubility of the inhibitor is sufficient in the critical temperature range of hydrate formation^23,24^. In fact, increasing the length of hydrocarbon chain, branching the pendant chain and the steric hindrance effect play a vital role in the kinetic inhibition mechanism of KHIs. Continuing with our interest in the inhibition of hydrate formation by kinetic hydrate inhibitors, we attempted to develop a novel and powerful application of a special group of polymers based on waterborne called “waterborne-based polymers” for design and synthesis of KHIs in order to achieve the best performance in the hydrate formation inhibition (Fig. 1). These waterborne poly urea/urethanes (WPUUs) were synthesized by a simple method, since available monomers and the final polymer do not need any purification. Also, as polyurethanes represent a group of polymers with very high demand in industries, they may be used as a new class of commercial KHIs. Given that the conventional products of polyurethanes (PUs) generally contain a considerable amount of organic solvents and free isocyanate monomers, and concerns about volatile organic chemicals (VOCs) and hazardous air pollutants (HAPs), waterborne polyurethanes (WPUs) have attracted more attention as one of the greenest replacement because of their eco-friendly and non-toxic nature. In comparison with production method of PUs, WPUs are produced at least of amount of VOCs during their curing process. Moreover, they can be modified easily^25–27^. Waterborne polymers have several advantages. Firstly, they are hydrophilic and hygroscopic and these features help them to create a homogeneous phase with water. In addition, they have functional groups such as urethane or hydroxyl groups and they can form intermolecular hydrogen bonds with the water molecules. The last but not least is that, these polymers with linear or cyclic alkyl groups can create a gap among water molecules and lead to disruption of hydrogen bonds among them. Thus, they can prevent hydrate formation successfully. Ionic liquids also have some of these features similar to waterborne, but there are two limitations in their synthesis. One limitation is that many of them are expensive, corrosive and not biocompatible. A second weakness, these compounds usually have low molecular weight^28–30^. In this work we introduce a new application of waterborne technique for synthesis of KHIs in order to use a variety of polar and non-polar groups for getting the polymer structure with expected properties. This technique not only has no limitation on the solubility in water, but it also will provide reasonable performance for KHIs.

## Results and Discussion

### High-pressure autoclave cell

#### Gas uptake experiments

**Figure 2:**
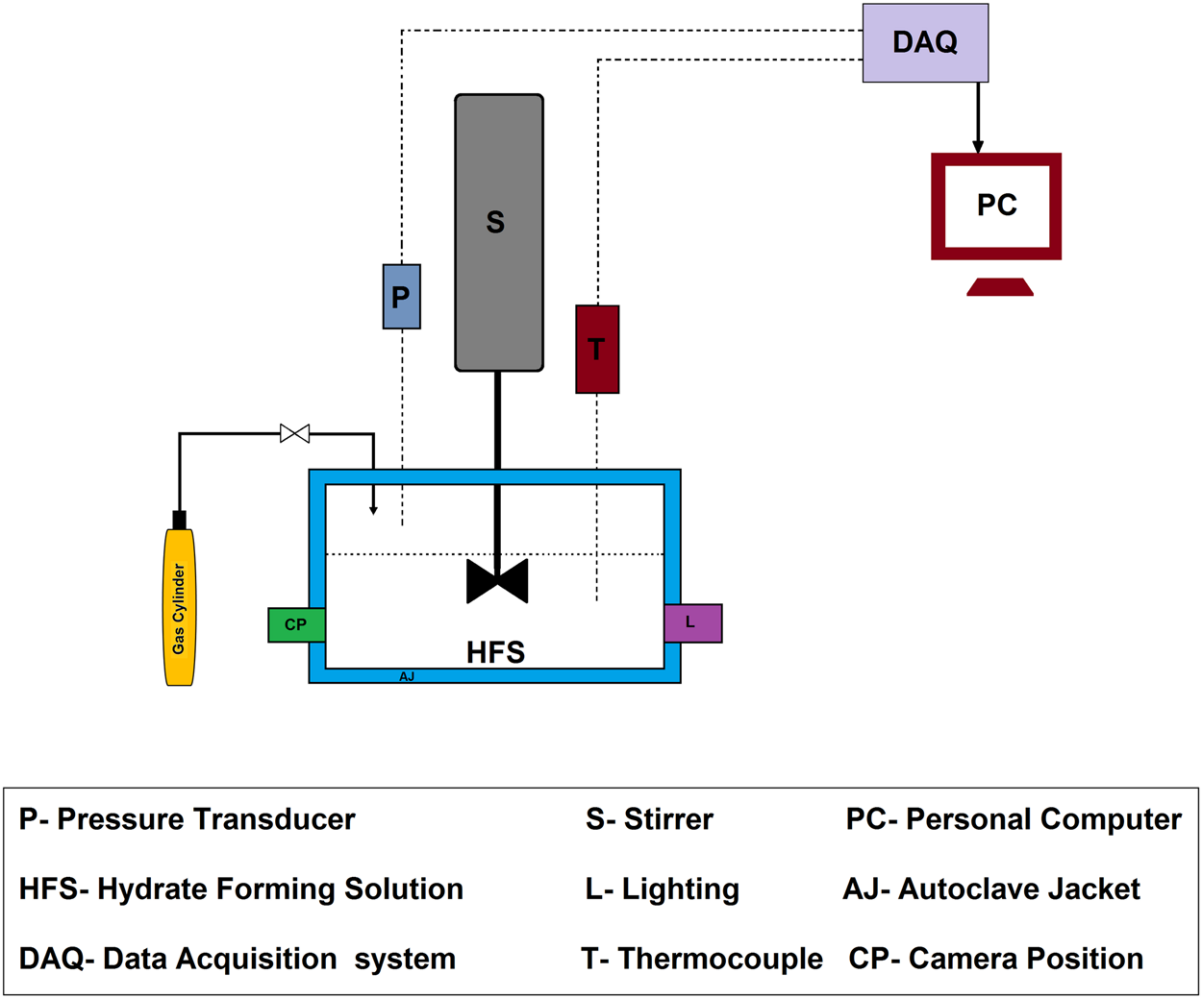
Schematic of the high-pressure autoclave cell.

**Figure 3:**
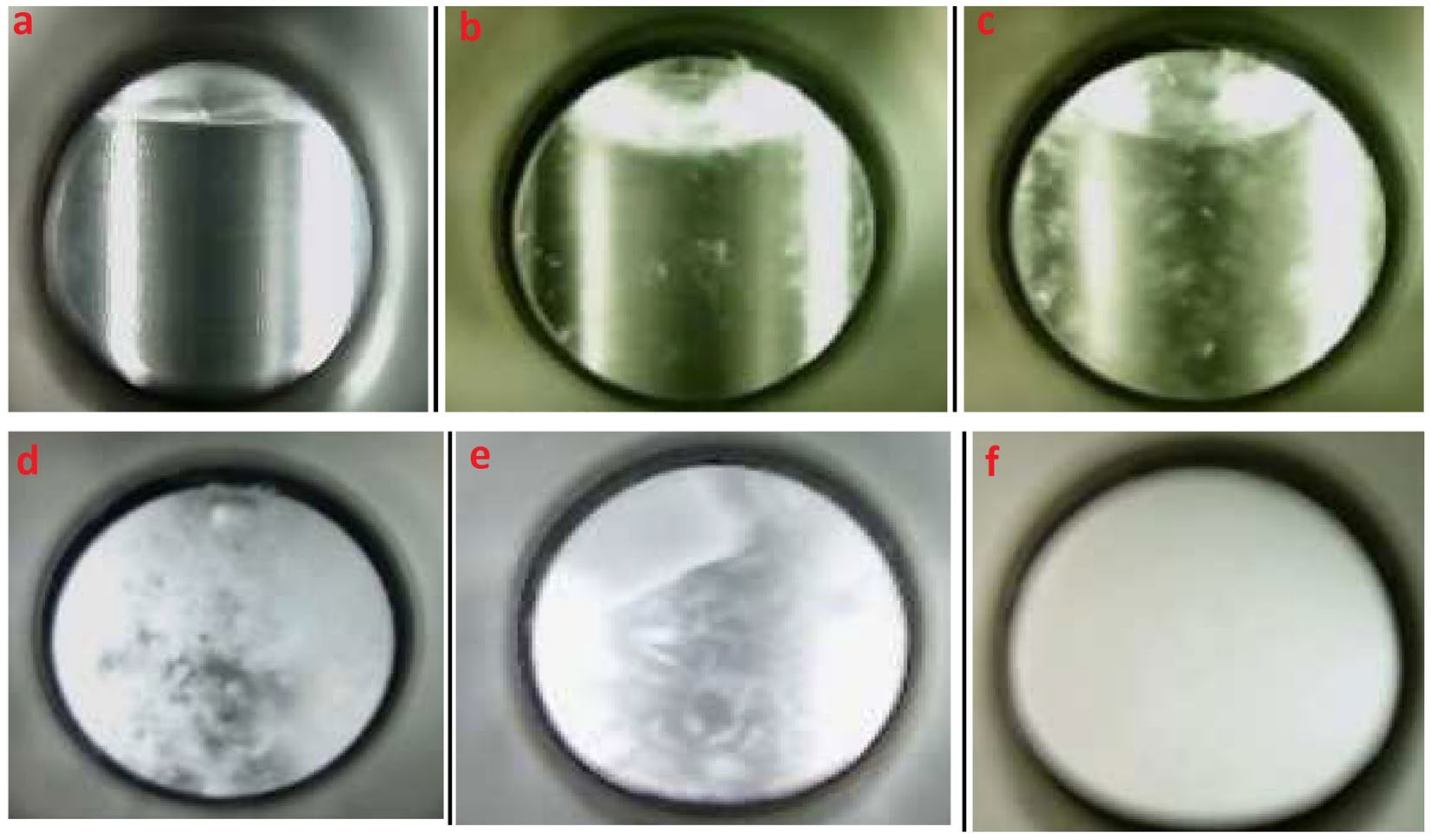
The macroscopic observation of the process of methane hydrate formation inside the high-pressure autoclave cell (**a**) the initial time of the reaction, (**b**,**c**) induction time, (**d**,**e**) the stages of hydrate growth, (**f**) complete hydrate formation.

Methane hydrate formation was evaluated by experiments performed in a high-pressure autoclave cell (Figs 2 and S5) and results summarized in Table 1. The induction time was marked as time period before fast pressure decrease, which indicates the growth of methane hydrates. This process was visible through a sapphire window and recorded by a camera (Fig. 3). Considerable delay in the induction time was observed in the presence of both Isophorone diisocyanate (IPDI) and hexamethylene diisocyanate (HDI)-based waterborne poly urea/urethane (WPUU) inhibitors (Table 1). As can be seen, these results show that both IPDI inhibitors with ~3.8 kD and ~1.7 kD molecular weights are effective kinetic inhibitors since they delayed hydrate nucleation by an average of around 40 and 30 min (in 1 wt% samples), respectively, as compared to water, in which hydrates nucleated in ~2 min. It wasreported^31^ that intermolecular forces between KHIs and nucleation sites are critical. However, inhibitors with capability to form a layer on the surface of a nucleus have a great effect in inhibiting hydrate formation. It was proposed that KHIs adsorb to heterogeneous nucleation sites in the aqueous phase and as a result delay hydrate nucleation^31,32^. With regard to this interpretation, it can be said that functional groups of WPUUs (urethane, urea or hydroxyl groups) can form intermolecular hydrogen bonds with the water molecules. In other words, these interactions dramatically reduced the number of hydrogen bonds among water molecules. As a result, a strong interaction with nucleation sites leads to significantly inhibit hydrate nucleation compared to pure water. It should also be noted that, as in the case of cubic structure I (sI) of methane gas hydrates, it was reported that strength of inhibitors is dependent on molecular weight of the polymer; lower molecular weight KHIs displayed better strength of inhibition^33^. In this study it is also clear that WPUUs with the medium and low molecular weight can cause more delay in the hydrate nucleation compared to a high molecular weight WPUU at the same conditions. This is probably due to the fact that, according to the perturbation mechanism for inhibiting nucleation WPUU with lower molecular weight might have better mobility in the water phase due to its low viscosity. Thus, the low molecular weight polymer is more successful in disrupting the water structure compared to higher molecular weight polymers. In fact, alongside several parameters such as steric hindrance and power of hydrogen bonding, optimum molecular weight of polymers has a significant effect for kinetic hydrate inhibition. Another interesting point should be noticed in Table 1: IPDI-based WPUU has better nucleation inhibition performance compared to HDI-based WPUU. Probably it can be said that in the IPDI-based WPUU the presence of cyclic groups in the back-bone of polymer instead of hexamethylene groups in the HDI-based WPUU can lead to more disruption in the local water structure. Hence it is also quite clear that, IPDI-based WPUU was a more effective inhibitor than HDI-based WPUU since it delayed nucleation by a factor of 14.87 and HDI-based WPUU delayed by a factor of 10.99 (in 1 wt% low molecular weight samples) in comparison with pure water. In these conditions (2 °C and 9 MPa) the induction time for water + Anti-Freeze Protein (AFP) systems formed with AFP-III and AFP-I was reported as 8 and 24 min, respectively^34^. Also it is reported that, in 274.15 K and 70 bar PVCap delayed nucleation time by a factor of 8.7 in comparison with pure water^35^. It is clear that, the temperature of hydrate nucleation in the presence of WPUUs are significantly delayed compared to the AFP-III, AFP-I and PVCap.

**Table 1:** Experimental conditions and induction times for methane gas hydrate formation at 2 °C and 9 MPa (standard uncertainties *u* are *u*(T) = 0.1 °C, *u*(P) = 0.005 MPa).

CH_4_ (99.95%)	No.	Concentration (wt%)	Induction time (min)	Mean induction time, ${\bar{{\bf{t}}}}_{{\bf{ind}}}$ (min)
Water	1	—	2	2.01
	2	—	2.14	
	3	—	1.9	
IPDI-based waterborne (∼1.7 kD)	4	0.1	3.6	3.2
	5		2.9	
	6		3.1	
	7	0.5	10.5	10.16
	8		9.3	
	9		10.7	
	10	1	32.4	29.9
	11		28	
	12		29.3	
IPDI-based waterborne (∼3.8 kD)	13	0.1	4	4
	14		4.2	
	15		3.8	
	16	0.5	11.25	12.32
	17		13.5	
	18		12.22	
	19	1	44	40.23
	20		37	
	21		39.7	
IPDI-based waterborne (∼7.2 kD)	22	0.1	3.3	2.83
	23		2.7	
	24		2.5	
	25	0.5	9.25	9.18
	26		8.2	
	27		10.1	
	28	1	25	22.36
	29		19.8	
	30		22.3	
HDI-based waterborne (∼2.1 kD)	31	0.1	2.8	2.53
	32		2.2	
	33		2.6	
	34	0.5	8.7	7.96
	35		7.3	
	36		7.9	
	37	1	20.9	22.1
	38		23.6	
	39		21.8

#### Hydrate growth

**Figure 4:**
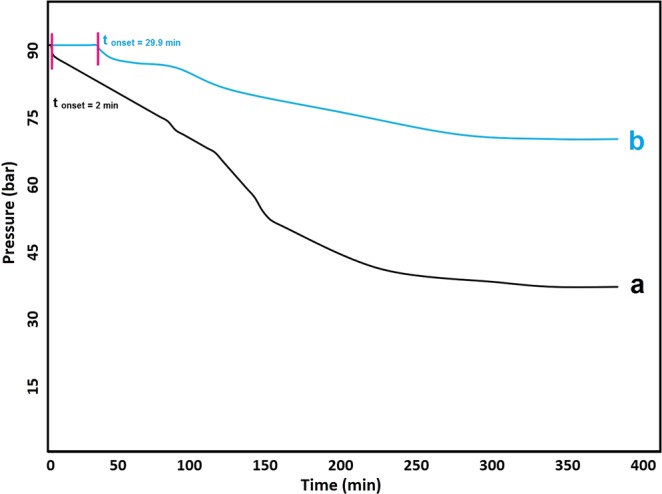
Hydrate formation profiles during autoclave (pressure drop) experiments at 2 °C: (**a**) pure water and (**b**) IPDI-based WPUU ~1.7 kD (in 1wt% samples).

As seen in Fig. 4, growth of methane hydrates after ~360 min in reactor was observed by a decrease in pressure from 9 to ~3.6 MPa in pure water, but this was reduced only from 9 to 7 MPa in the presence of IPDI-based WPUU ~1.7 kD (in 1 wt% samples). This means that, in pure water system after 360 min ~60 wt% of methane converted to hydrate, while this value for aqueous solution of IPDI-based WPUU is ~22 wt%. These results clearly show that IPDI-based WPUU inhibitor helps to reduce hydrate growth significantly. It is believed, that developed KHIs can reduce hydrate growth by two mechanisms, including adsorption–inhibition and perturbation inhibition^31^. Therefore, we propose that the reason for the variations in hydrate formation kinetics due to different inhibitors can be a difference in the perturbation of the water structure or in the adsorption to nascent hydrate crystals. Thus, these inhibitors not only have good solubility in water, but also functional groups of these inhibitors efficiently adsorb into the hydrate surface and disrupt the water structure. Thus, they are able to cause significant delay in the formation of hydrates. It should be noted that, it has been reported in previous works of different authors^36,37^ that in laboratory experiments KHIs can induce significant increase of methane hydrates growth that has been called “catastrophic hydrate growth”. This may be a critical problem if it occurs in a field application of KHIs. However, for inhibitors synthesized in this work the catastrophic hydrate growth is not observed (as seen in Fig. 4). They show opposite effect of decreasing the hydrate growth in three times comparing with pure water.

### High pressure micro-differential scanning calorimeter (HP-μDSC)

#### Hydrate formation

**Figure 5:**
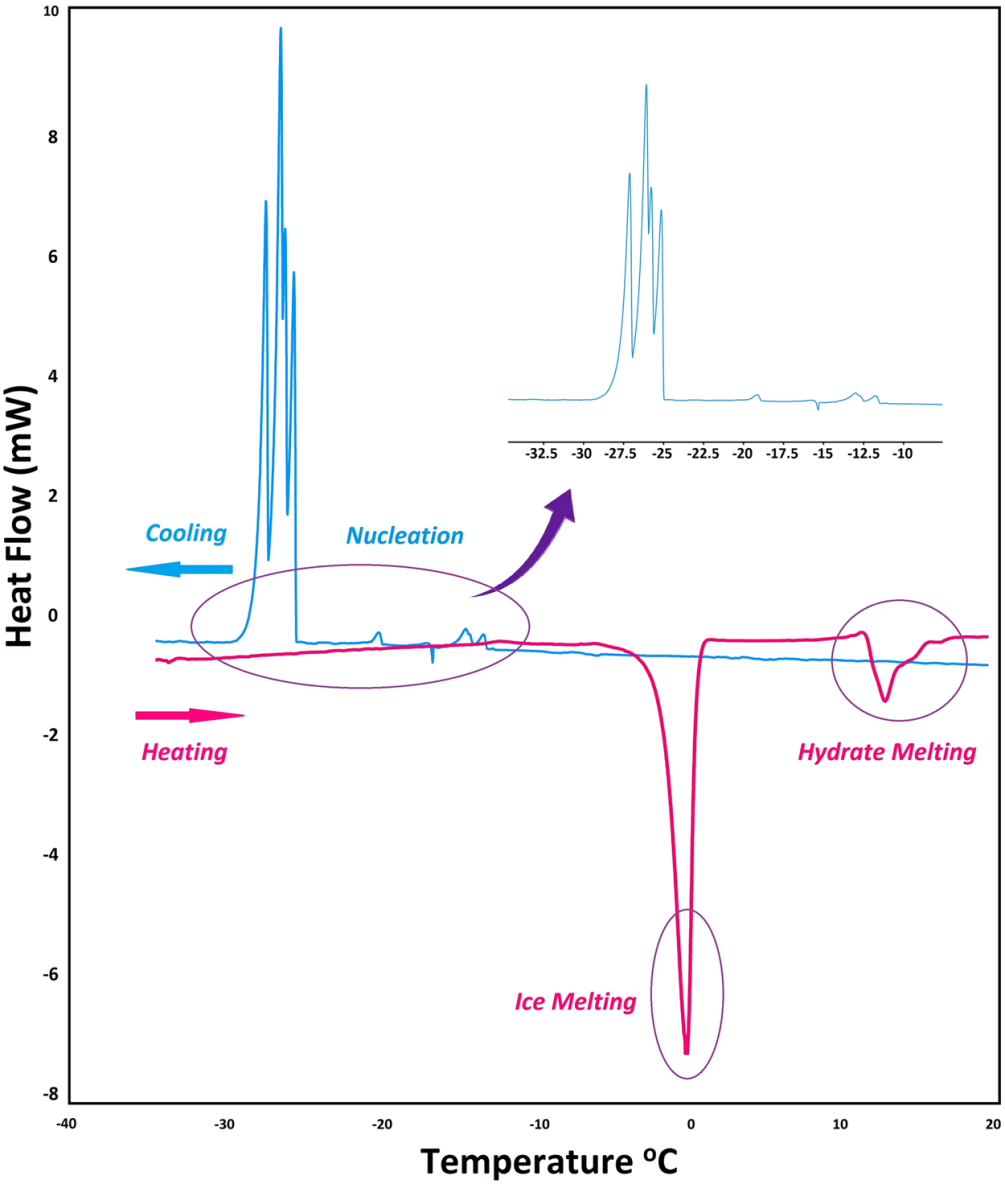
Typical DSC cooling and heating curves (pure water) obtained using the temperature ramping method in which the temperature of the samples is dropped from +20 to −35 °C and then reheated, all at 9 MPa.

**Figure 6:**
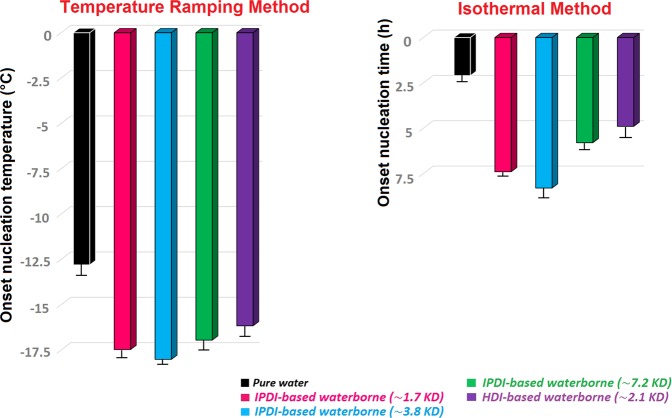
Onset nucleation temperature and time in fresh water (with/without inhibitor) during temperature ramping and isothermal experiments (number of experiments = 3, and error bars represents standard deviation (SD)).

DSC as a convenient tool to investigate hydrate formation/dissociation was used to determine the onset time/temperature of hydrate formation, as well as thermal behavior of hydrates formed (Fig. S5). Figure 5 demonstrates a typical DSC thermogram for methane + water system in the ramping method. In this condition, the onset nucleation temperature can be determined by the temperature at which the first peak in the curve is observed (See Fig. 5). During the cooling period, two peaks were observed that are related to the hydrate and ice formation^38^. In contrast to water molecules, methane molecules have less contact with water and as a result the amount of ice formed should be greater than hydrate^39^. This interpretation is compatible with the integrated area of the hydrate exotherm that is smaller than ice exotherm. On the other hand, the melting behavior confirms that the hydrate formation is less than the amount of ice formed. As seen in Fig. 5, during ramping runs when pure water was cooled from 20 to −35 °C, four separate exothermic peaks related to hydrate/ice and nucleation were observed in four different capillaries (circled as nucleation). Then with increasing temperature to 20 °C, two distinct endothermic peaks were observed (Fig. 5, circled as ice and hydrate melting). Ices formed were melted at ~0 °C and hydrate melting peak was observed at ~12 °C. Figure 6 summarizes the onset nucleation temperature during cooling in the ramping experiments in the presence of WPUUs. In the fresh water the onset nucleation temperature was observed at around −12.8 °C. Hence, the performance of an inhibitor depends on the amount of delay in the onset nucleation temperature. From Fig. 6 it can be clearly deduced that, WPUUs are able to decrease the average onset nucleation temperature from −12.8 °C to −18.01 °C depending on the type of WPUUs that have been used. To further confirm the accuracy of the results of autoclave tests, isothermal experiment with DSC was used to determine the induction time. Figure 6 summarizes the results of the onset nucleation time in the isothermal tests in the pure water and WPUU systems. The results of Fig. 6 show that, the onset induction time for pure water is 2.1 hour, while the presence of WPUUs increased the average onset induction time from 2.1 to 8.3 hour depending on the type of WPUUs that have been used. Similar to the autoclave results in the DSC isothermal tests, IPDI-based waterborne (~3.8 kD) showed the best performance in increasing the induction time. These results are comparable with the work of Daraboina *et al*.^40^, where in the presence of Luvicap the average onset induction time was increased from 2.3 (in pure water system) to ~6.2 hours. Also the similar trends in nucleation delay in the presence of these types of inhibitors were also seen in the high pressure autoclave experiments. It has been reported that, the performance of poly vinyl caprolactam (PVCap) is related to the molecular weight of the polymer. On the other hand, it is suggested that, the optimum molecular weight (Mw) for a KHI polymer to achieve the best performance is around 1500–3000. A possible explanation for the favorable molecular weight range is that the surface/volume ratio of the polymer can lead to the maximum perturbation of the surrounding water and the high mobility of the polymer in solution^32^. The results of this work demonstrates that, in contrast to high molecular weight WPUU the sample with the medium molecular weight have better nucleation inhibition performance at the same conditions and also both of them have better performance than Luvicap-EG (low Mw polyvinyl caprolactam as a 41% solution in ethylene glycol solvent)^40^. Similar to the high-pressure autoclave experiments, these results show that these WPUUs are strong nucleation inhibitors. In fact, design/synthesis of new inhibitors with optimal molecular weight is one of the most striking differences between waterborne technique and ionic liquids.

#### Hydrate decomposition

**Figure 7:**
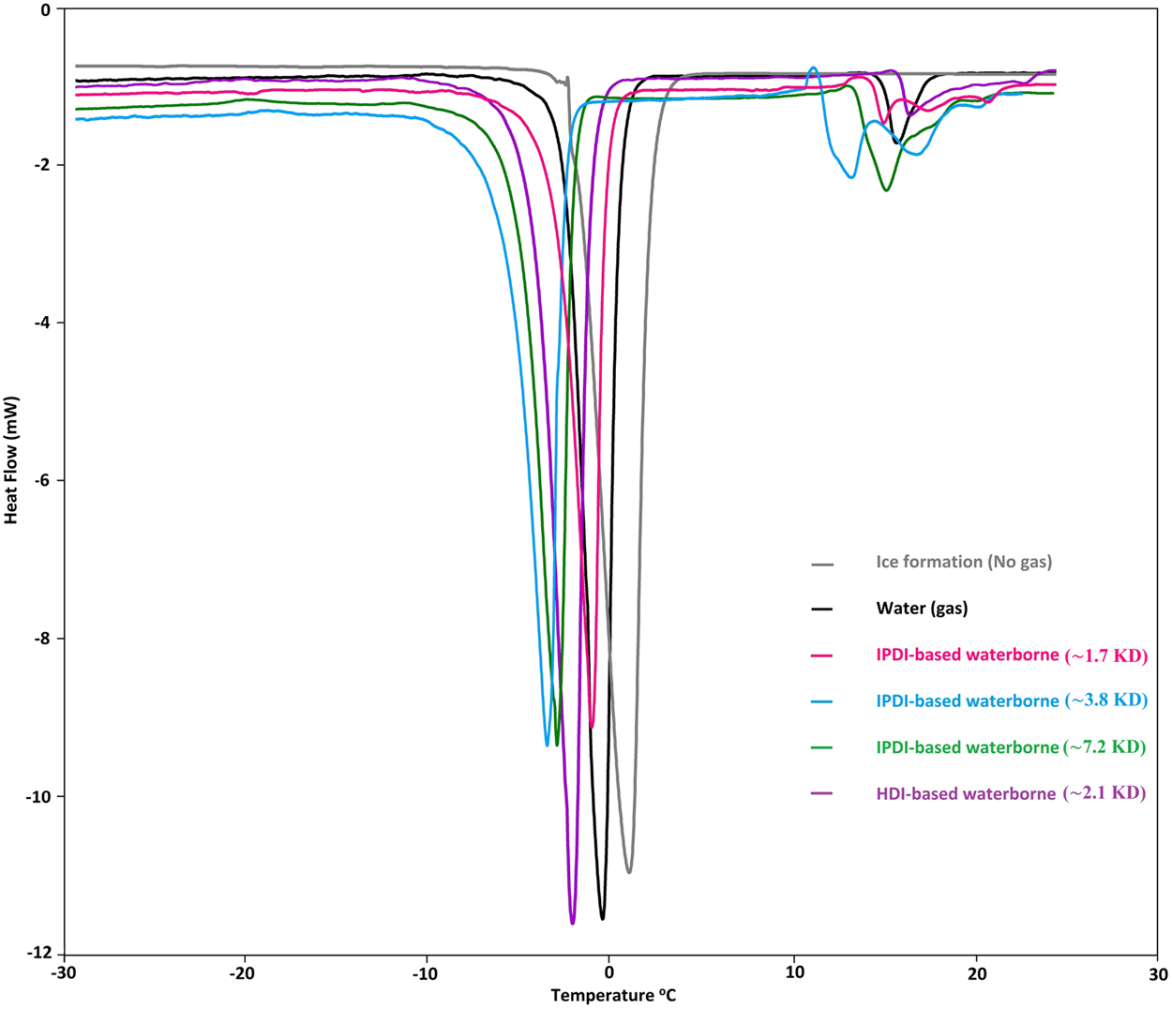
Typical hydrate melting curves in the presence or absence of WPUUs at 9 MPa using the temperature ramping protocol.

To investigate the melting behavior of hydrate formed in temperature ramping the temperature was raised at 0.25 °C/min up to 20 °C. Figure 7 shows the ice/hydrate melting peaks for water, IPDI/HDI-based waterborne solution during the heating cycle. As can be seen clearly, the multiple hydrate melting peaks were obtained with WPUUs. This is due to the fact that the hydrate formed in the presence of WPUUs was heterogeneous (the multiple hydrate melting peaks) in comparison with the homogeneous (the single peak) melting observed with water sample. It has been reported that, kinetic inhibitors such as PVCap leading to a higher dissociation temperature compared to hydrates formed in the uninhibited systems, or that they increased the time required for complete hydrate decomposition^41,42^. As a result, further energy is needed to eliminate hydrate plugs formed under conditions where these kinetic inhibitors are applied, even at their lowest concentrations^41–44^. Two possible reasons have been proposed for higher hydrate dissociation temperature in the presence of KHIs. Firstly, the formation of more hydrates and secondly, increased hydrate stability, which then takes longer to melt. Contrary to this, the amount of hydrates formed in the presence of WPUU is much less compared to pure water (seen in Fig. 4). On the other hand, decreasing in the hydrate dissociation temperature has been achieved in the presence of WPUUs and the final dissociation point has remained almost unchanged (Fig. 7). So, it can be concluded that, these new KHIs not only can delay the induction time and reduce the hydrate growth rate, but also they would not increase hydrate dissociation temperature in comparison with pure water. Hence, this is a valuable and promising result for KHIs field applications. Moreover, the decomposition data for all KHIs based on IPDI clearly indicate thermodynamic inhibition. Decomposition temperature of methane gas hydrates in presence of proposed KHIs is lower than in pure water.

## Conclusions

In this study, we report for the first time the use of waterborne polymers as a novel class of powerful inhibitors to prevent the formation of methane hydrates. The performance of these new KHIs as inhibitors was investigated by high pressure micro-differential scanning calorimeter (HP-μDSC) and high-pressure autoclave cell. The results of both analyses demonstrated the high performance of these inhibitors in delaying the induction time and reducing the hydrate growth rate. Also in contrast with pure water, for inhibitors synthesized in this work the catastrophic hydrate growth is not observed and they would not increase hydrate dissociation temperature. This is the first report of the use of waterborne polymers as kinetic hydrate inhibitor and more research is needed in different conditions (specially for sII hydrates) to improve their performance. We believe that these results will provide new insight in synthesis/design of KHIs with high performance. Currently, our group is investigating the performance of waterborne polymers as anti-agglomerant hydrate inhibitors. This technique can inspire new ideas for synthesizing novel KHIs with higher inhibition performance.

## Experimental Section

### Materials

Isophorone diisocyanate (IPDI) and hexamethylene diisocyanate (HDI), polyethylene glycol 400 (PEG), triethylamine (TEA) and deuterated dimethyl sulfoxide-d_6_ (99.8% d) were purchased from Sigma-Aldrich. 2,2-Bis(hydroxymethyl)propionic acid (DMPA), ethanolamine (EA) and tetrahydrofuran were obtained from Merck Chemical Co. All reagents were used without further purification. Methane gas of 99.95% purity was used for experiments. Water used for methane hydrates formation experiments and for preparation of inhibitors solution of desired concentration was carefully purified. On the first step it was twice distillated. On the second step it was deionized using Arium mini plus ultra-pure water system (Sartorius, Germany) to achieve resistivity 18.20 MΩ·cm at 25 °C.

### Characterization methods

NMR spectra were recorded on a Bruker Avance400-MHz. The spectra were recorded with a frequency of 400 MHz by using DMSO at room temperature. The chemical shifts were referenced to the solvent signals. Infrared spectra (600–4000 cm^−1^) were obtained using a Vertex 70 FTIR spectrometer (Bruker, Germany) equipped with single reflection ZnSe crystal ATR accessory (MIRacle, PIKE Technologies). Background spectra of 128 scans at a resolution of 4 cm^−1^ were subtracted from the sample spectra. The data was processed through program OPUS 7.2 (Bruker).

#### Synthesis of waterborne poly urea/urethane (WPUU)

Waterborne poly urea/urethanes (WPUU)s were synthesized without using any catalyst and Fig. 1 shows the procedure and conditions of synthesis of (WPUU). Briefly, the IPDI or HDI, PEG 400 and DMPA were added to a three-necked flask powered by a mechanical stirrer, nitrogen inlet, condenser, and thermometer. Table S1 summarized the molar ratio between the NCO groups of the IPDI or HDI, the OH groups of the PEG 400, and the OH groups of the DMPA. The reaction mixture was heated at 70 °C for 30 min to obtain a homogeneous mixture because it is critical for the resulting identical uniform distribution of hydrophilic monomer, DMPA, on the polyurethane main chain. After that, the reaction was followed at 85 °C for 2 hours under a dry nitrogen atmosphere, and 30 ml of THF was added to reduce the viscosity of the system. After 2 hours, EA was added to the system and the reaction was continued for 5 hours at 70 °C. After polymerization, the reaction mixture was cooled to ambient temperature and then neutralized by the addition of TEA (1.2 equivalent per DMPA), followed by dispersion at high speed with deionized water to prepare the WPUU (Fig. S1). It should be noted that, although polyurethanes are non-toxic, but they can produce toxic substances during their degradation process^45^. ^1^H NMR (400 MHz, DMSO-d6) δ 7.34–6.83 (m, 1H), 5.87 (s, 0H), 4.10–3.96 (m, 3H), 3.62–3.50 (m, 7H), 3.51 (s, 22H), 3.42 (t, J = 5.0 Hz, 1H), 3.04 (p, J = 5.9 Hz, 1H), 2.72 (s, 1H), 2.68 (q, J = 6.9 Hz, 1H), 2.09 (s, 3H), 1.45 (s, 3H), 1.08 (s, 1H), 1.02 (t, J = 5.9 Hz, 1H), 1.01–0.90 (m, 9H), 0.87 (t, J = 3.9 Hz, 5H), 0.82–0.74 (m, 2H). ^13^C NMR (101 MHz, DMSO-d6) δ 181.25, 158.94, 157.28, 155.83, 72.79, 70.23, 69.35, 64.46, 63.59, 63.37, 61.33, 60.66, 54.76, 53.70, 47.04, 45.94, 44.34, 42.51, 41.81, 40.19, 39.98, 39.78, 35.45, 31.86, 31.15, 30.38, 28.05, 27.95, 23.64, 23.57, 10.77.

### Apparatus and procedure

#### High-pressure autoclave cell

Figure 2 shows a schematic description of the high-pressure autoclave cell with sapphire windows (model 2929 0000, TOP INDUSTRIE, France). The gas uptake tests were carried out in a high-pressure autoclave cell to measure hydrate induction times in the pure water and WPUU solutions (Fig. S5). To measure the induction time of the WPUUs, we chose the detection of pressure difference as an indicator. The autoclave thermocouple and pressure sensor were calibrated by the producer. Also, standard uncertainties (*u*) of temperature and pressure measurements for this instrument were determined and were equal to *u*(T) = 0.1 °C and *u*(P) = 0.005 MPa. During the formation of gas hydrate, methane is encaged into the hydrate structure, therefore the system pressure decreases. A sudden pressure drop and temperature rise indicates the formation of gas hydrates. Thus, from the beginning of the test, the temperature and pressure were continuously monitored while gas hydrate formation occurred. To determine the nucleation delay time of methane hydrate formation, selected WPUU samples were prepared as an aqueous solution with a specified concentration. A volume of 30 ± 0.1 ml of solution was charged in a 30 ml autoclave cell that was prepared at 2 °C and under vacuum to avoid the possibility of remaining gases. Methane gas with a nominal purity of 99.95% was then injected into the cell at a pressure of 9 MPa. After approximately 20 minutes, the high-pressure autoclave cell was reached at 2 °C and the solution was stirred at 400 rpm with a magnetic spin bar during the whole experiment. The time zero was assigned at the point when the desired autoclave cell pressure and temperature had been reached. It is known that gas hydrate formation consists of three parts including, gas dissolution, nucleation and growth of crystals. Hence, we defined the induction times of the corresponding sample under conditions reached at the end of the dissolution step. After starting stirrer, the solution, the pressure of the methane and temperature were recorded. Once the formation of the hydrate began, an abrupt decrease in pressure was clearly seen as seen in Fig. 4. Pressure and time, at which hydrates started to form (Ts and Ps) were recorded and selected as the onset nucleation hydrate formation. Time from the beginning to Ts was chosen as “the induction time”. The induction time of each sample solution was measured during three parallel experiments and their scattering values due to the stochastic nature of nucleation were averaged. The induction time for hydrate formation in pure water measured at 2 °C and 9 MPa in our work (which is equal to 2 minutes) coincides well with literature data (also is equal to 2 minutes)^34^, and confirm reliability of our measurements.

#### High pressure micro-differential scanning calorimeter (HP-μDSC)

A high-pressure micro differential scanning calorimeter SETARAM 7 evo (HP DSC) powered with two 1 ml cells stable to high pressure was applied to evaluate the thermal behavior of ice and hydrate (Fig. S5). A gas pressure panel was used to control the pressure. This panel has a piston which can be used to charge automatically the sample with methane at pressures ranging from 0.1 to 100 MPa. The μ-DSC can also be applied to investigate thermal behaviors of hydrates and ice at these pressures and at temperatures between −45 °C and 120 °C. The thermal sensors in the calorimetric furnace directly measure the heat flow exchanged between the reference and sample cells and the temperature-programmed furnace. The instrument was calibrated using standard sample of pure naphthalene provided by Setaram. The melting temperature (80.3 ± 0.1 °C) and the enthalpy of fusion (19.01 ± 0.09 kJ mol^−1^) of naphthalene measured in this work were in excellent agreement with recommended literature data *T*_*m*_ = 80.4 °C and Δ_*fus*_*H*_*m*_ = 19.06 ± 0.08 kJ mol^−1^ ^46^. This fact confirms the reliability of heat and temperature values measurements by our calorimeter. To increase the surface area (or to overcome the mass transfer resistance) in DSC cell, water or aqueous solution (2 μl) was added into capillaries (2.3 mm diameter and 0.9 cm length, four in our case) with a micro syringe according to method proposed previously^40^. Capillary tubes were then placed inside the HP-μDSC cell and each test was reproduced three times. To supply the required test pressure methane was injected in the sample cell and afterward temperature programs were started. DSC analysis was used to measure onset nucleation temperature/time using the temperature ramping and isothermal modes. Hence, after the pressure inside the cell was reached 90 bar, ramping tests were carried from 20 to −35 °C then was raised from −35 to 20 °C at a heating/cooling rate of 0.25 °C/min. On the other hand, to determine the hydrate nucleation onset time isothermal method was applied. In this method the temperature was decreased quickly from 25 °C to −12 °C at 1 °C/min and was fixed at −12 °C for 10 hours. The onset nucleation processes were detected as starting temperature of exothermic peaks with evolution of time at −12 °C. After 10 hours, the temperature was increased similar to ramping method to record hydrate melting. Three experiments were performed for each sample with 1 wt%.

## Supplementary information

Supporting Information

## References

[1] Sloan, E. D. J. & Koh, C. Clathrate hydrates of natural gases 3^rd^ edn, (CRC Press, 2007).

[2] Kang KC, Linga P, Park K-n, Choi SJ, Lee JD (2014). Seawater desalination by gas hydrate process and removal characteristics of dissolved ions (Na+, K+, Mg2+, Ca2+, B3+, Cl−, SO42−). Desalination.

[3] Veluswamy HP, Kumar R, Linga P (2014). Hydrogen storage in clathrate hydrates: Current state of the art and future directions. Appl. Energy.

[4] Zhong D, Englezos P (2012). Methane Separation from Coal Mine Methane Gas by Tetra- n -butyl Ammonium Bromide Semiclathrate Hydrate Formation. Energy Fuels.

[5] Kumar A, Kumar R (2015). Role of Metallic Packing and Kinetic Promoter in Designing a Hydrate Based Gas Separation Process. Energy Fuels.

[6] Wang W, Bray CL, Adams DJ, Cooper AI (2008). Methane storage in dry water gas hydrates. J. Am. Chem. Soc..

[7] Sugahara T (2009). Increasing Hydrogen Storage Capacity Using Tetrahydrofuran. J. Am. Chem. Soc..

[8] Chapoy A, Anderson R, Tohidi B (2007). Low-Pressure Molecular Hydrogen Storage in Semi-clathrate Hydrates of Quaternary Ammonium Compounds. J. Am. Chem. Soc..

[9] Kim E, Ko G, Seo Y (2017). Greenhouse Gas (CHF 3) Separation by Gas Hydrate Formation. ACS Sustain. Chem. Eng..

[10] Wang W (2014). Methane storage in tea clathrates. Chem. Commun..

[11] Arora A (2016). Biosurfactant as a Promoter of Methane Hydrate Formation: Thermodynamic and Kinetic Studies. Sci. Rep..

[12] Linga P, Clarke MA (2017). A review of reactor designs and materials employed for increasing the rate of gas hydrate formation. Energy Fuels.

[13] Kelland MA (2006). History of the Development of Low Dosage Hydrate Inhibitors. Energy Fuels.

[14] Perrin A, Musa M, Steed JW, Perrin A, Musa OM (2013). The chemistry of low dosage clathrate hydrate inhibitors. Chem. Soc. Rev..

[15] Xu S (2016). Pectin as an Extraordinary Natural Kinetic Hydrate Inhibitor. Sci. Rep..

[16] Sa, J., Kwak, G., Lee, B. R., Han, K. & Lee, K. Hydrophobic amino acids as a new class of kinetic inhibitors for gas hydrate formation. *Sci. Rep*. 1–7, 10.1038/srep02428 (2013). 10.1038/srep02428 PMC3741619 23938301

[17] Dong S, Firoozabadi A (2015). Hydrate anti-agglomeration and synergy effect in normal octane at varying water cuts and salt concentrations. J. Chem. Thermodyn..

[18] Dong S, Li M, Firoozabadi A (2017). Effect of salt and water cuts on hydrate anti-agglomeration in a gas condensate system at high pressure. Fuel.

[19] Ke W, Kelland MA (2016). Kinetic Hydrate Inhibitor Studies for Gas Hydrate Systems: A Review of Experimental Equipment and Test Methods. Energy Fuels.

[20] Qin H-B (2015). Synthesis and Evaluation of Two New Kinetic Hydrate Inhibitors. Energy Fuels.

[21] Lin H, Wolf T, Wurm FR, Kelland MA (2017). Poly(alkyl ethylene phosphonate)s: A New Class of Non-amide Kinetic Hydrate Inhibitor Polymers. Energy Fuels.

[22] Magnusson CD, Kelland MA (2015). Nonpolymeric Kinetic Hydrate Inhibitors: Alkylated Ethyleneamine Oxides. Energy Fuels.

[23] Magnusson CD, Liu D, Chen EY-X, Kelland MA (2015). Non-Amide Kinetic Hydrate Inhibitors: Investigation of the Performance of a Series of Poly(vinylphosphonate) Diesters. Energy Fuels.

[24] Abrahamsen E, Kelland MA (2016). Carbamate Polymers as Kinetic Hydrate Inhibitors. Energy Fuels.

[25] Zhou X (2015). Recent Advances in Synthesis of Waterborne Polyurethane and Their Application in Water-based Ink: A Review. J. Mater. Sci. Technol..

[26] Hu J (2016). Click crosslinking improved waterborne polymers for environment-friendly coatings and adhesives. ACS Appl. Mater. Interfaces.

[27] Shendi HK (2017). Synthesis and characterization of a novel internal emulsifier derived from sunflower oil for the preparation of waterborne polyurethane and their application in coatings. Prog. Org. Coatings.

[28] Kim, K. *et al*. Ionic liquids based on N-alkyl-N-methylmorpholinium salts as potential electrolytes. *Chem. Commun. (Camb)*. 828–9, 10.1039/b400198b (2004). 10.1039/b400198b 15045084

[29] Tariq M (2014). Gas Hydrate Inhibition: A Review of the Role of Ionic Liquids. Ind. Eng. Chem. Res..

[30] Kim K, Kang JW, Kang S (2011). Tuning ionic liquids for hydrate inhibition. Chem. Commun..

[31] Zeng H, Wilson LD, Walker VK, John A (2003). The inhibition of tetrahydrofuran clathrate-hydrate formation with antifreeze protein. Can. J. Phys..

[32] Daraboina N, Ripmeester J, Walker VK, Englezos P (2011). Natural Gas Hydrate Formation and Decomposition in the Presence of Kinetic Inhibitors. 3. Structural and Compositional Changes. Energy Fuels.

[33] Sloan ED, Subramanian S, Matthews PN, Lederhos JP, Khokhar AA (1998). Quantifying Hydrate Formation and Kinetic Inhibition. Ind. Eng. Chem. Res..

[34] Daraboina N, Moudrakovski IL, Ripmeester JA, Walker VK, Englezos P (2013). Assessing the performance of commercial and biological gas hydrate inhibitors using nuclear magnetic resonance microscopy and a stirred autoclave. Fuel.

[35] Kang S-P (2013). Unusual synergy effect on methane hydrate inhibition when ionic liquid meets polymer. RSC Adv..

[36] Cha M, Shin K, Seo Y, Shin JY, Kang SP (2013). Catastrophic growth of gas hydrates in the presence of kinetic hydrate inhibitors. J. Phys. Chem. A.

[37] Sharifi H, Englezos P (2015). Accelerated hydrate crystal growth in the presence of low dosage additives known as kinetic hydrate inhibitors. J. Chem. Eng. Data.

[38] Handa YP (1986). Compositions, enthalpies and heat capacities in the 270 K for clathrate hydrates and dissociation of isobutane determined by a heat-flow of dissociation, range 85 to of methane, enthalpy of hydrate, as calorimeter. J. Chem. Thermodyn..

[39] Mochizuki T, Mori YH (2006). Clathrate-hydrate film growth along water / hydrate-former phase boundaries — numerical heat-transfer study. J. Cryst. Growth.

[40] Daraboina N, Malmos C, Solms NV (2013). Investigation of Kinetic Hydrate Inhibition Using a High Pressure. Energy Fuels.

[41] Gulbrandsen AC, Svartaas TM (2017). Effect of Poly Vinyl Caprolactam Concentration on the Dissociation Temperature for Methane Hydrates. Energy Fuels.

[42] Lachance JW, Sloan ED, Koh CA (2009). Determining gas hydrate kinetic inhibitor effectiveness using emulsions. Chem. Eng. Sci..

[43] Sharifi H, Ripmeester J, Englezos P (2016). Recalcitrance of gas hydrate crystals formed in the presence of kinetic hydrate inhibitors. J. Nat. Gas Sci. Eng..

[44] Lee JD, Englezos P (2006). Unusual kinetic inhibitor effects on gas hydrate formation. Chem. Eng. Sci..

[45] Prisacariu, C. Polyurethane Elastomers From Morphology to Mechanical Aspects. (Springer-Verlag/Wien, 2011).

[46] Roux MV, Temprado M, Chickos JS, Nagano Y (2008). Critically evaluated thermochemical properties of polycyclic aromatic hydrocarbons. J. Phys. Chem. Ref. Data.

