# Supplementary material for: Waterborne Polyurethanes as a New and Promising Class of Kinetic Inhibitors for Methane Hydrate Formation
Source: Sci Rep. 2019 Jul 5;9:9797. doi: 10.1038/s41598-019-46274-w (PMC6611824; doi:10.1038/s41598-019-46274-w)
Supplement: Supplementary file 1 — Supporting Information [file 41598_2019_46274_MOESM1_ESM.docx]

**Waterborne Polyurethanes as a New and Promising Class of Kinetic Inhibitors for Methane Hydrate Formation**

*Abdolreza Farhadian^1^, Arman Kudbanov^1^, Mikhail A. Varfolomeev^1^*, Didier Dalmazzone^2^*

^1^Department of Physical Chemistry, Kazan Federal University, Kremlevskaya str. 18, 420008 Kazan, Russian Federation.

^2^UCP, ENSTA ParisTech, Université Paris-Saclay, 828 Boulevard des Maréchaux, 91762 Palaiseau Cedex, France.

* Corresponding Author E-mail address: [mikhail.varfolomeev@kpfu.ru](mailto:mikhail.varfolomeev@kpfu.ru).

Table S1. Chemical composition of synthesized waterborne poly urea/urethanes (WPUU)s

| Sample | NCO (mol) | PEG 400 (mol) | DMPA (mol) | EA (mol) | TEA (mol) | Molecular weight (kD) |
| --- | --- | --- | --- | --- | --- | --- |
| IPDI-based WPUU | 0.0153 | 0.0084 | 0.0053 | 0.0015 | 0.00636 | ∼1.7 |
| IPDI-based WPUU | 0.0306 | 0.01692 | 0.0107 | 0.003 | 0.01284 | ∼3.8 |
| IPDI-based WPUU | 0.0612 | 0.0338 | 0.0204 | 0.006 | 0.02448 | ∼7.2 |
| HDI-based WPUU | 0.0306 | 0.01692 | 0.0107 | 0.003 | 0.0214 | ∼2.1 |


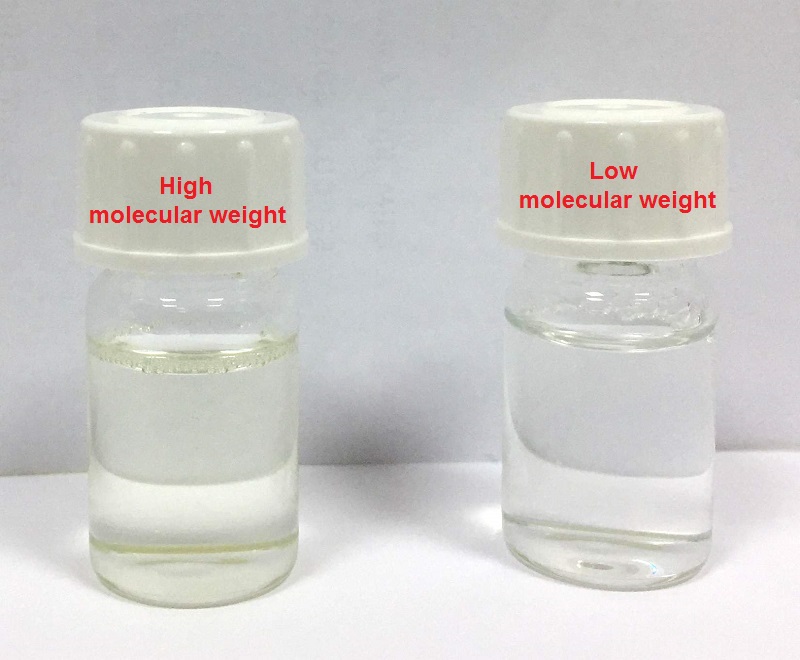


Figure S1: waterborne poly urea/urethane solution


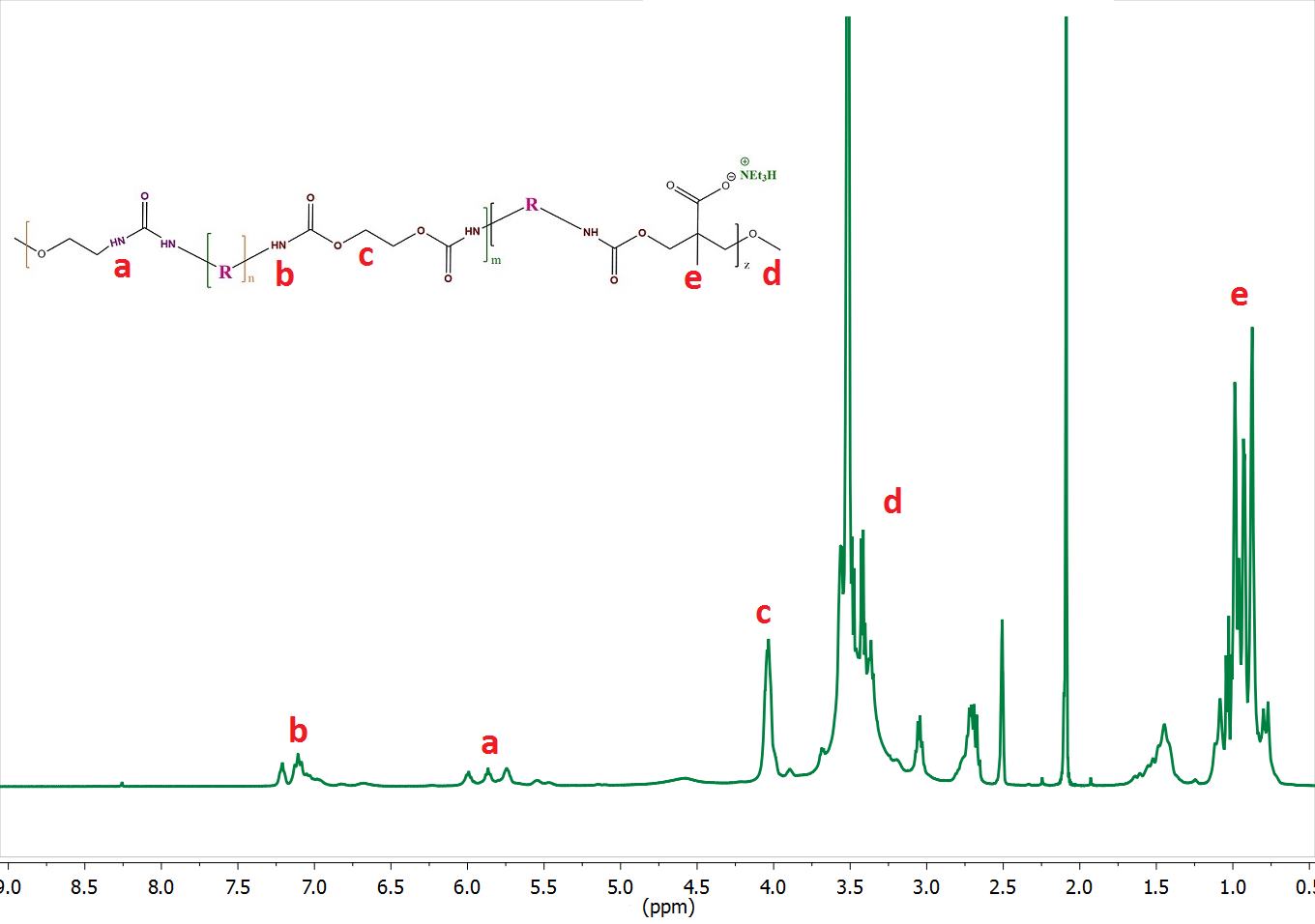


Figure S2: ^1^H NMR spectra of IPDI-based waterborne poly urea/urethane (IPDI-WPUU)


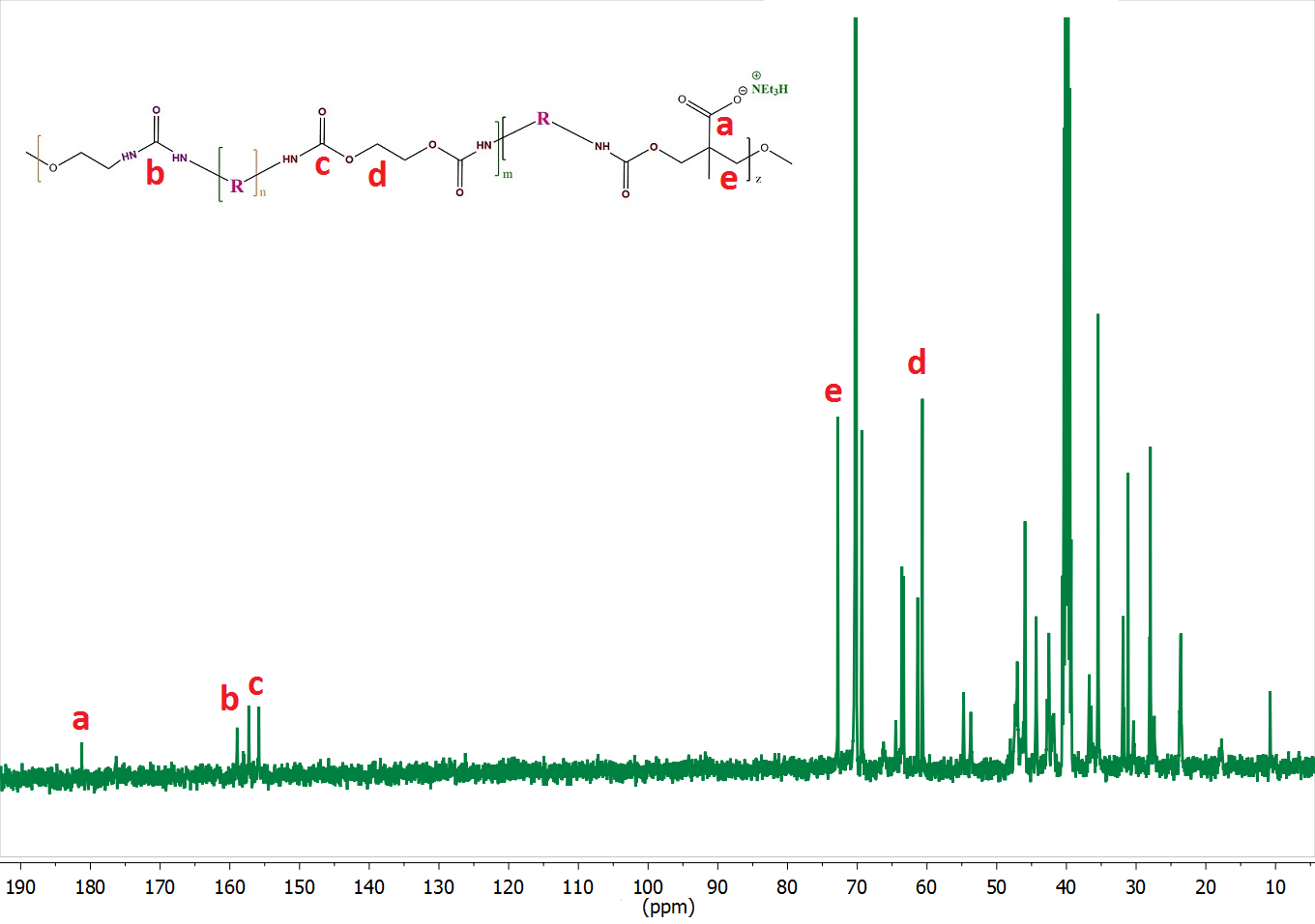


Figure S3: ^13^C NMR spectra of IPDI-based waterborne poly urea/urethane (IPDI-WPUU)


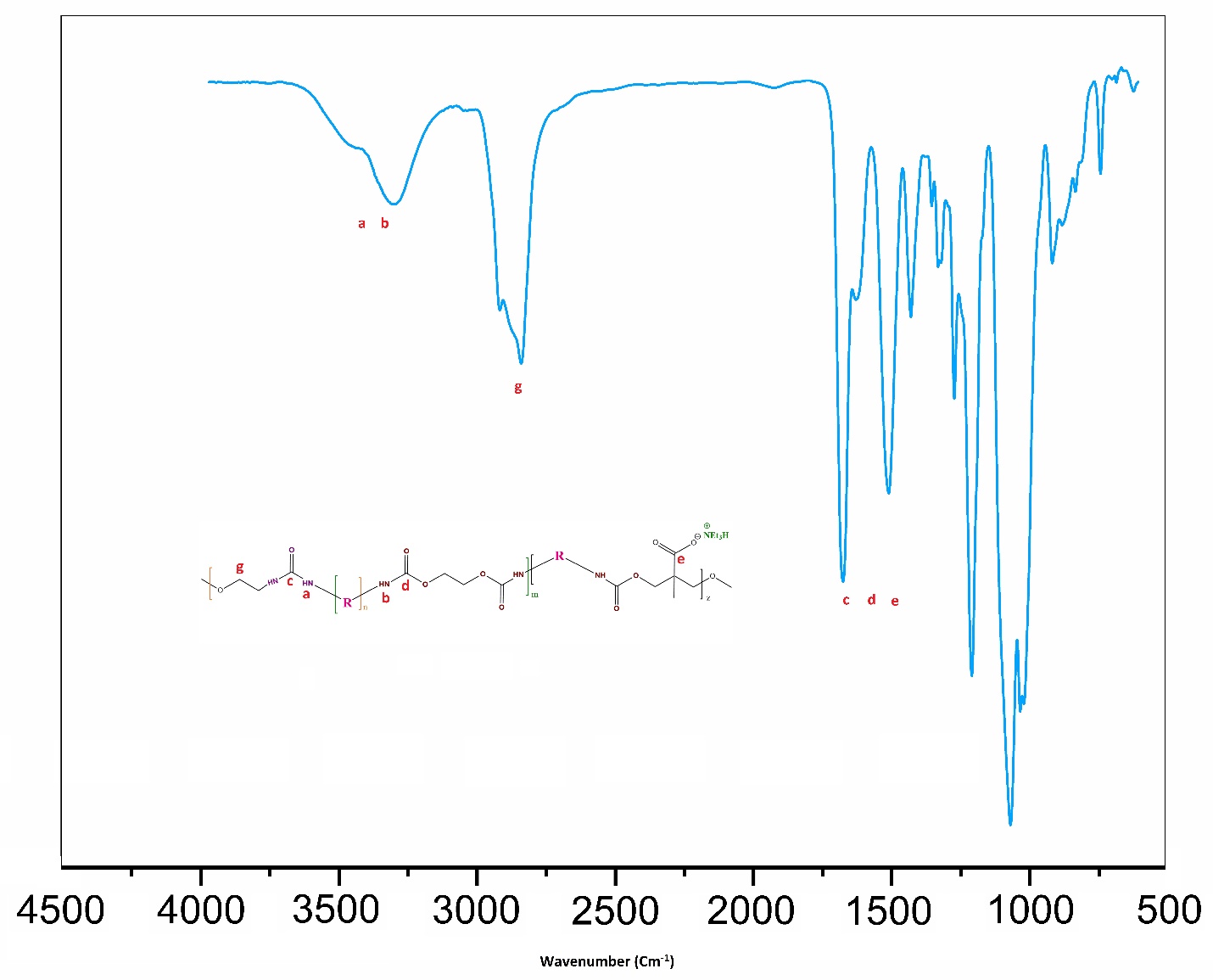


Figure S4: FT-IR spectra of IPDI-based waterborne poly urea/urethane (IPDI-WPUU)


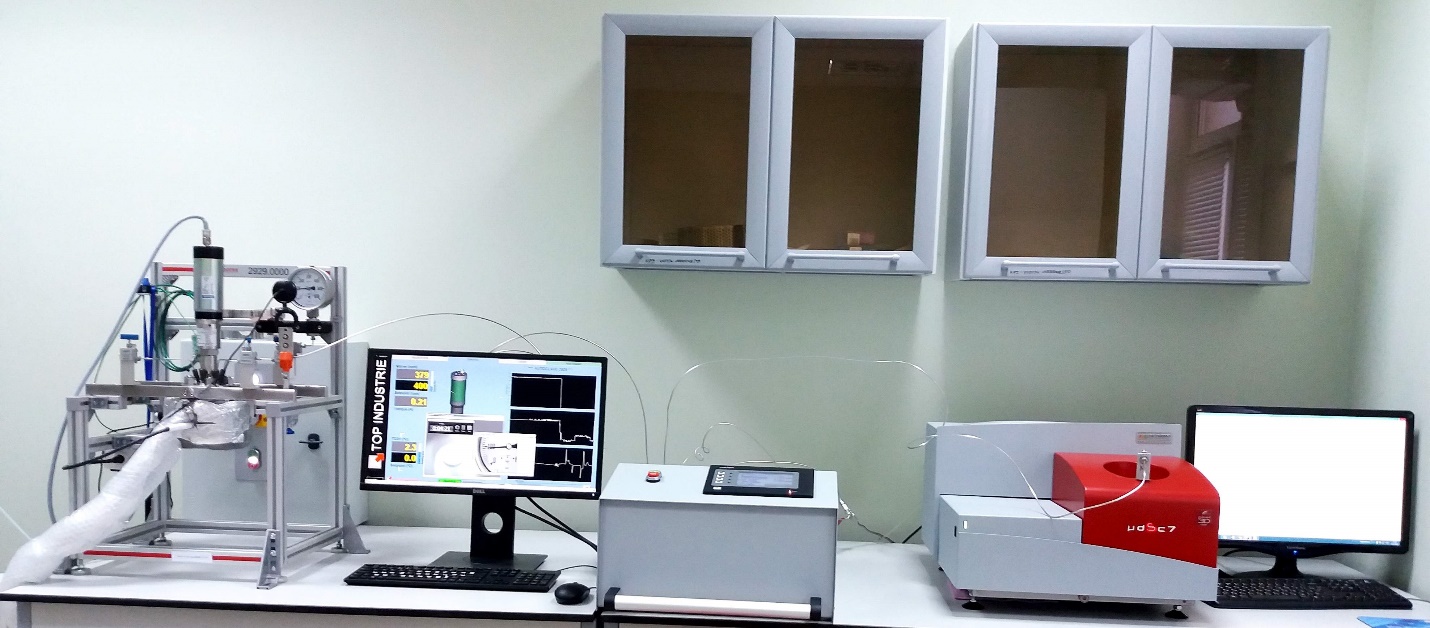


Figure S5. High-pressure autoclave cell and High pressure micro-differential scanning calorimeter (HP-μDSC)
